# Supplementary material for: Methylation of histone H4 lysine 20 by PR-Set7 ensures the integrity of late replicating sequence domains in Drosophila
Source: Nucleic Acids Res. 2016 Apr 29;44(15):7204–18. doi: 10.1093/nar/gkw333 (PMC5009726; doi:10.1093/nar/gkw333)
Supplement: SUPPLEMENTARY DATA [file supp_44_15_7204__index.html]

Methylation of histone H4 lysine 20 by PR-Set7 ensures the integrity of late replicating sequence domains in Drosophila — SUPPLEMENTARY DATA 

# Methylation of histone H4 lysine 20 by PR-Set7 ensures the integrity of late replicating sequence domains in *Drosophila*

## SUPPLEMENTARY DATA

- SUPPLEMENTARY DATA
