# Supplementary material for: Methylation of histone H4 lysine 20 by PR-Set7 ensures the integrity of late replicating sequence domains in Drosophila
Source: Nucleic Acids Res. 2016 Apr 29;44(15):7204–18. doi: 10.1093/nar/gkw333 (PMC5009726; doi:10.1093/nar/gkw333)
Supplement: SUPPLEMENTARY DATA [file supp_gkw333_nar-03357-m-2015-File003.pdf]

Figure S1. Overexpression of WT and PIP-mutant PR-Set7. (A) PR-Set7^{WT} or PR-Set7^{PIPm} cells were treated with HU and copper sulfate for 24 hours and harvested for the analysis of PR-Set7 levels by immunoblotting (Orc6 serves as a loading control). (B) Cell cycle profiles by flow cytometry after overexpression of asynchronous PR-Set7^{WT} or PR-Set7^{PIPm} for 72 hours.

Figure S2. PR-Set7 depletion is sufficient to prevent the accumulation of H4K20me1 upon release from a Dacapo-induced G1 cell cycle arrest. (A) Control and PR-Set7 depleted cells enter late S/G2 within 8 hours following release from a Dacapo-induced G1 arrest. (B) RNAi-mediated depletion of PR-Set7 is sufficient to block the monomethylation of H4K20 in late S/G2 following release from the Dacapo induced cell cycle arrest. Immunofluorescence of H4K20me1 (red) in control and PR-Set7 depleted cells 8 hours following release into S phase. DNA is stained with DAPI (blue). (C) Boxplots resulting from the quantification of H4K20me1 intensity for each condition (n>180).

Figure S3. H4K20me1 levels are globally increased throughout the genome in late S phase. (A) Cell sorting. Hoechst 33342 stained cells were sorted into G1/S and late S/G2 populations based on DNA content. (B) Enrichment of H4K20me1 (RPKM) over a representative 4 MB region of chromosome 2L for cells enriched in G1/early S (blue) or late S/G2 (orange) of the cell cycle by cell sorting. (C) H4K20me1 is enriched at transcription start sites and gene bodies. Aggregate plots of H4K20me1 enrichment in G1/early S (blue) and late S/G2 (orange) cells relative to transcription start sites. (D) H4K20me1 levels increase in both genic and intergenic regions during late S phase. Boxplots representing the distribution of H4K20me1 RPKM within 13428 genic and 11430 intergenic regions ($p<2.2\times10^{-16}$).

Figure S4. Verification of ATR and ATM RNAi efficacy. The cells were incubated with control, ATR, ATM, or both ATR and ATM dsRNA for 48 hours before 0.05% MMS was added and incubated for 2 hours (labeled as “MMS +”). The intensity of H2A.v phosphorylation (γ-H2A.v) in response to MMS treatment was measured by immunofluorescence microscopy and depicted with boxplots (n > 250).

Figure S5. BrdU incorporation in HU arrested cells is enriched at ORC binding sites. Heatmap of BrdU incorporation (measured as RPKM) in relation to 5159 ORC binding sites (41). Below the dashed line are the 1677 ORC sites at early origins and above the dashed line are ORC sites at late origins. The center of ORC peak is labeled as position 0.

Figure S6. Genome-wide distribution of γ-H2A.v for control and PR-Set7 depleted cells. ChIP-seq analysis of the genome-wide distribution of γ-H2A.v for control and PR-Set7 depleted cells (RPKM).

Figure S7. Loss of PR-Set7 induced DNA damage does not specifically localize to the heterochromatin. (A) Asynchronous cells treated with PR-Set7 RNAi for 72 hours before immunofluorescence staining of HP1 (red; Abcam ab24726, 1:250) and γ-H2A.v (green). DNA is counterstained with DAPI (blue). (B) γ-H2A.v ChIP-seq datasets analyzed in Fig 6 were re-aligned to include repetitive pericentric heterochromatic regions (Bowtie parameter: -M 1). The γ-H2A.v log2 difference was calculated as in Fig 6. Boxplots of the log2 difference in γ-H2A.v signal between control and PR-Set7 RNAi depleted cells in early euchromatic replicating domains, late euchromatic replicating domains, and heterochromatic regions are shown.

Figure S8. ATR co-depletion reduces γ-H2A.v caused by loss of PR-Set7. Asynchronous cells were treated with control, PR-Set7, ATR, and PR-Set7/ATR RNAi for 72 hours and harvested for immunofluorescence staining with antibodies against γ-H2A.v. The γ-H2A.v intensity was determined and depicted in boxplots for each condition ($n > 1100$).

Figure S9. Disruption of the homologous recombination (HR) pathway does not reduce DNA damage resulting from loss of PR-Set7. Cells were treated with control or PR-Set7 RNAi for 72 hours before harvest and immunofluorescence staining of γ-H2A.v. The immunofluorescence intensity of γ-H2A.v under each condition was depicted with boxplots ($n > 250$).

Table S1. List of the chromosomal positions of late replicating domains and γ-H2A.v domains identified by a hidden Markov model.

Table S2. List of primer sequences used for generating dsRNAs.

Table S3. List of biological replicates for H4K20me1 ChIP-seq, origin activity, and γ-H2A.v ChIP-seq genomic assays and the pearson correlations between individual replicate datasets.

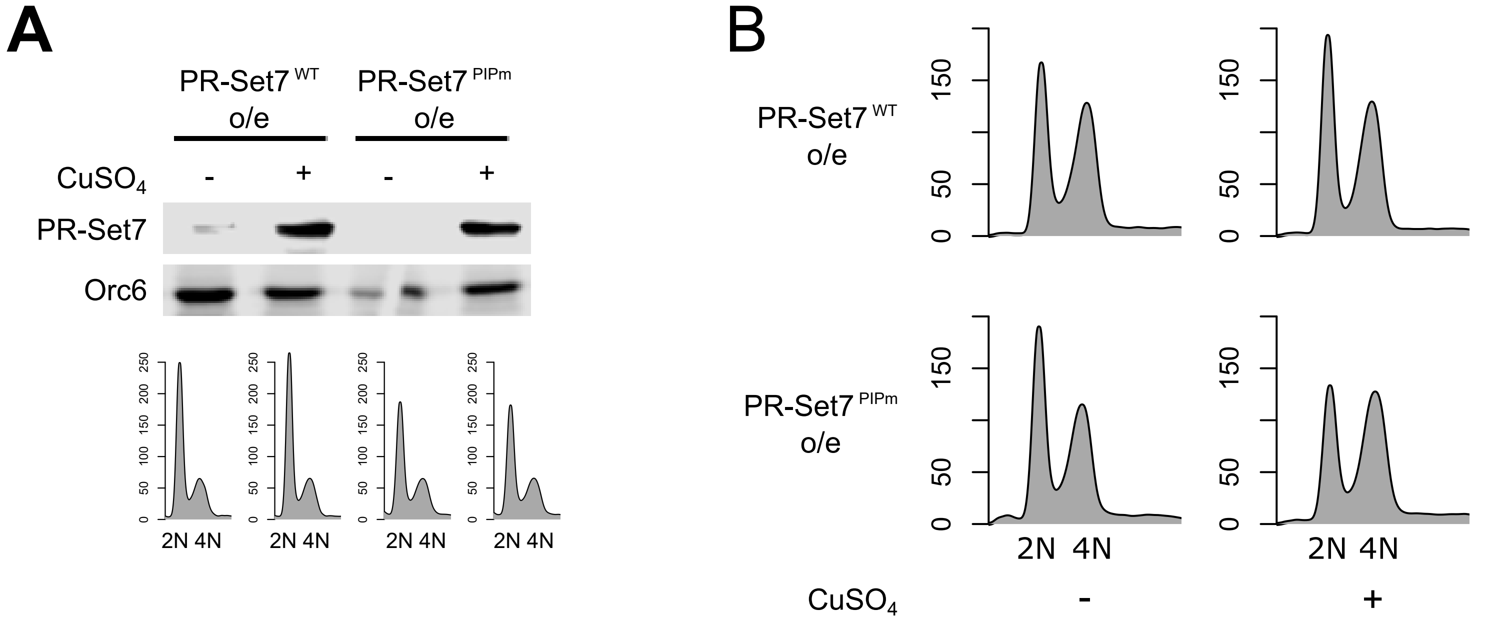
A
PR-Set7 WT o/e
PR-Set7 PIPm o/e
CuSO4
-
+
-
+
PR-Set7
Orc6
2N 4N
B
PR-Set7 WT o/e
PR-Set7 PIPm o/e
0
50
150
2N 4N
CuSO4
-
+

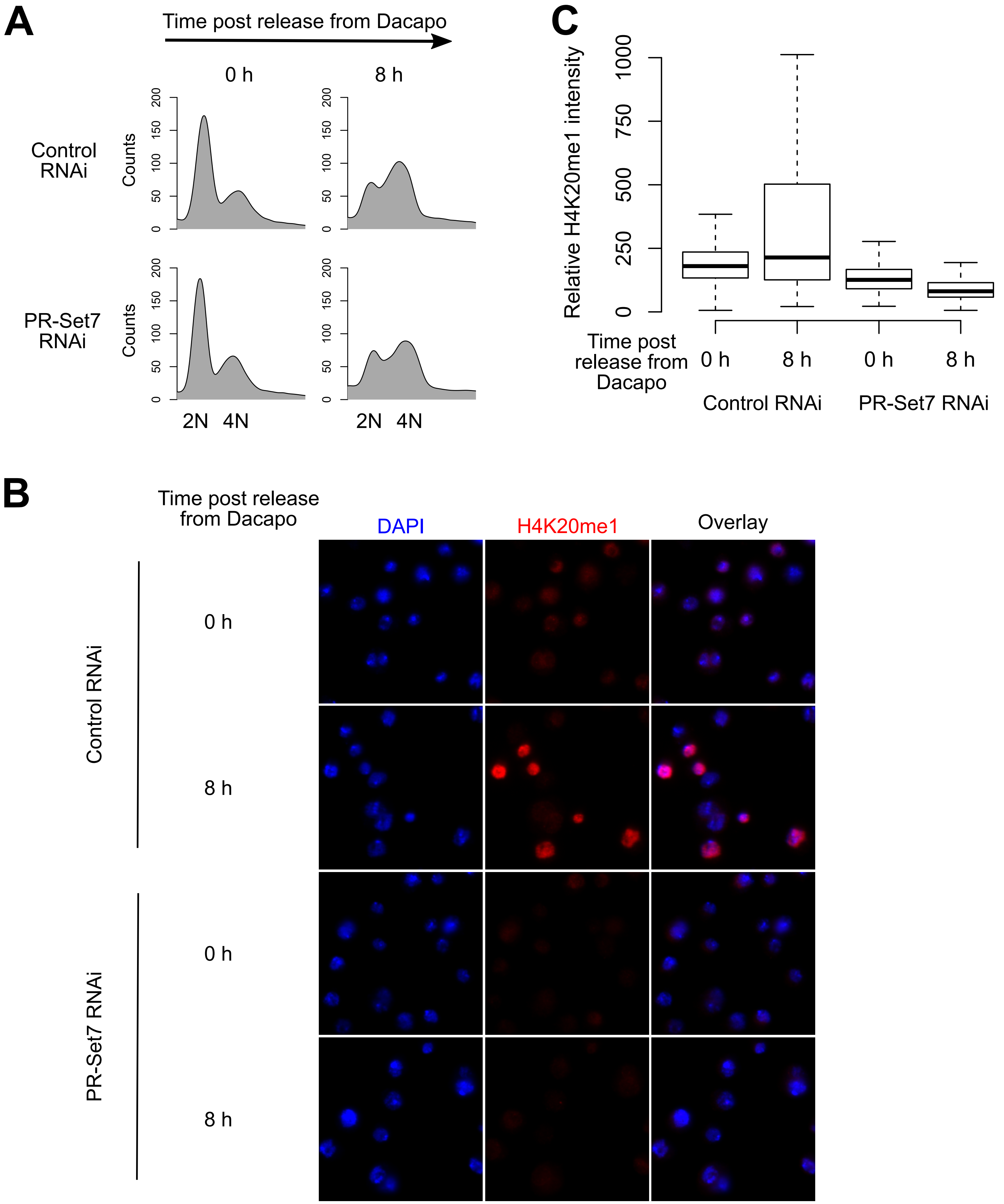
A
Time post release from Dacapo
0 h
8 h
Control RNAi
PR-Set7 RNAi
Counts
0
50
100
150
200
2N
4N
B
Time post release from Dacapo
DAPI
H4K20me1
Overlay
Control RNAi
PR-Set7 RNAi
0 h
8 h
C
Relative H4K20me1 intensity
0
250
500
750
1000
Time post release from Dacapo
0 h
8 h
Control RNAi
PR-Set7 RNAi

Li et al., Fig S3

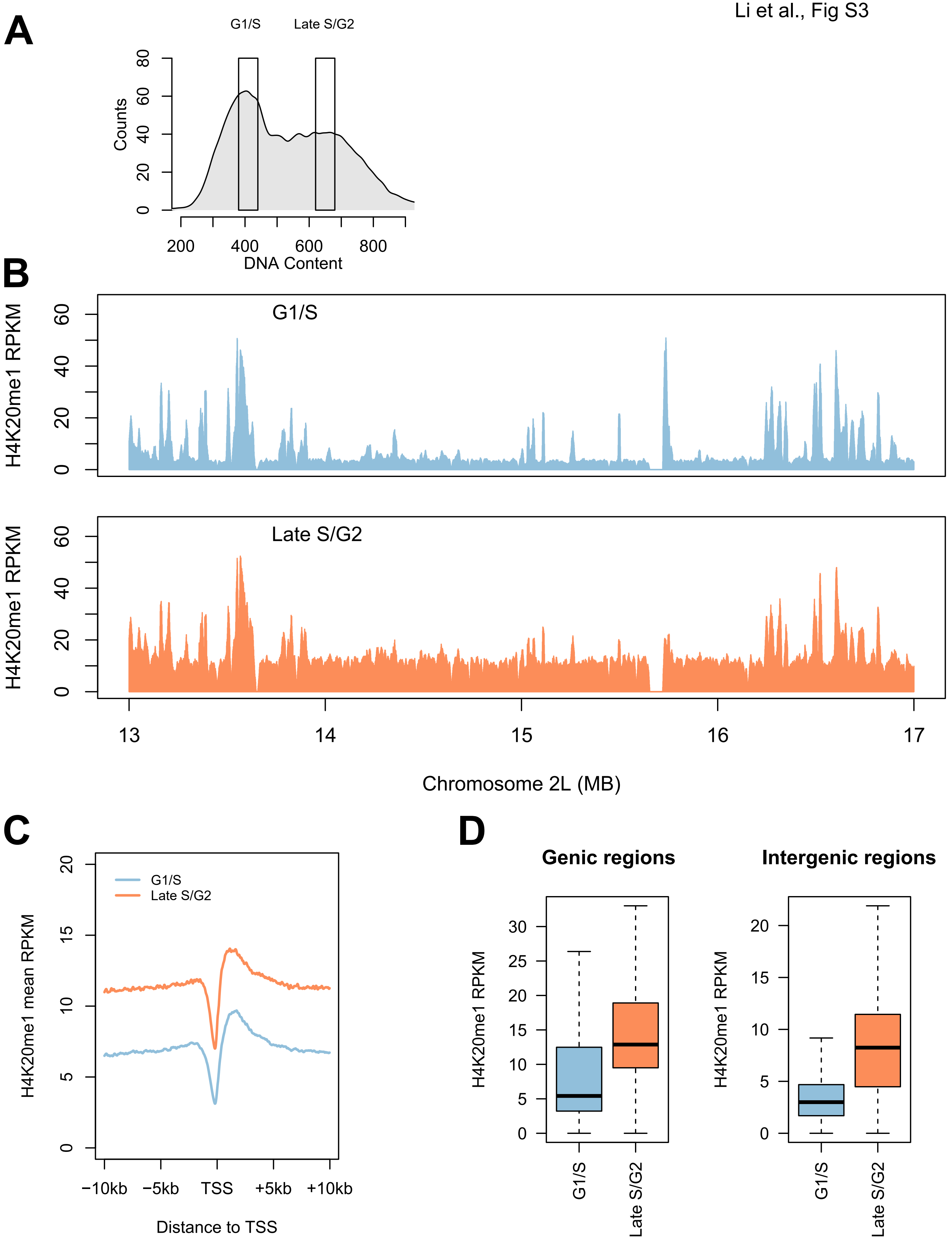

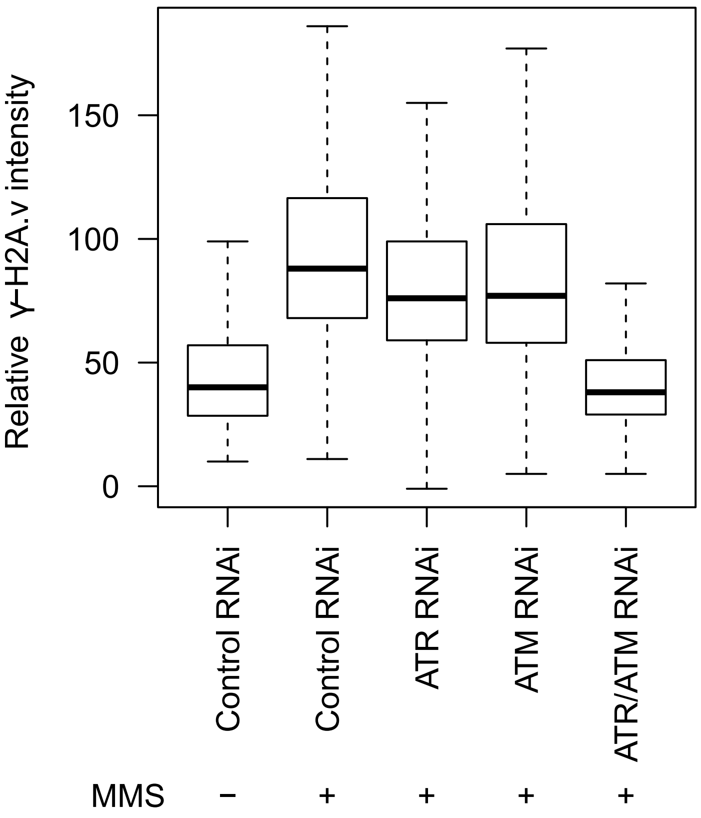
Relative γ-H2A.v intensity
0
50
100
150
Control RNAi
Control RNAi
ATR RNAi
ATM RNAi
ATR/ATM RNAi
MMS
−
+
+
+
+

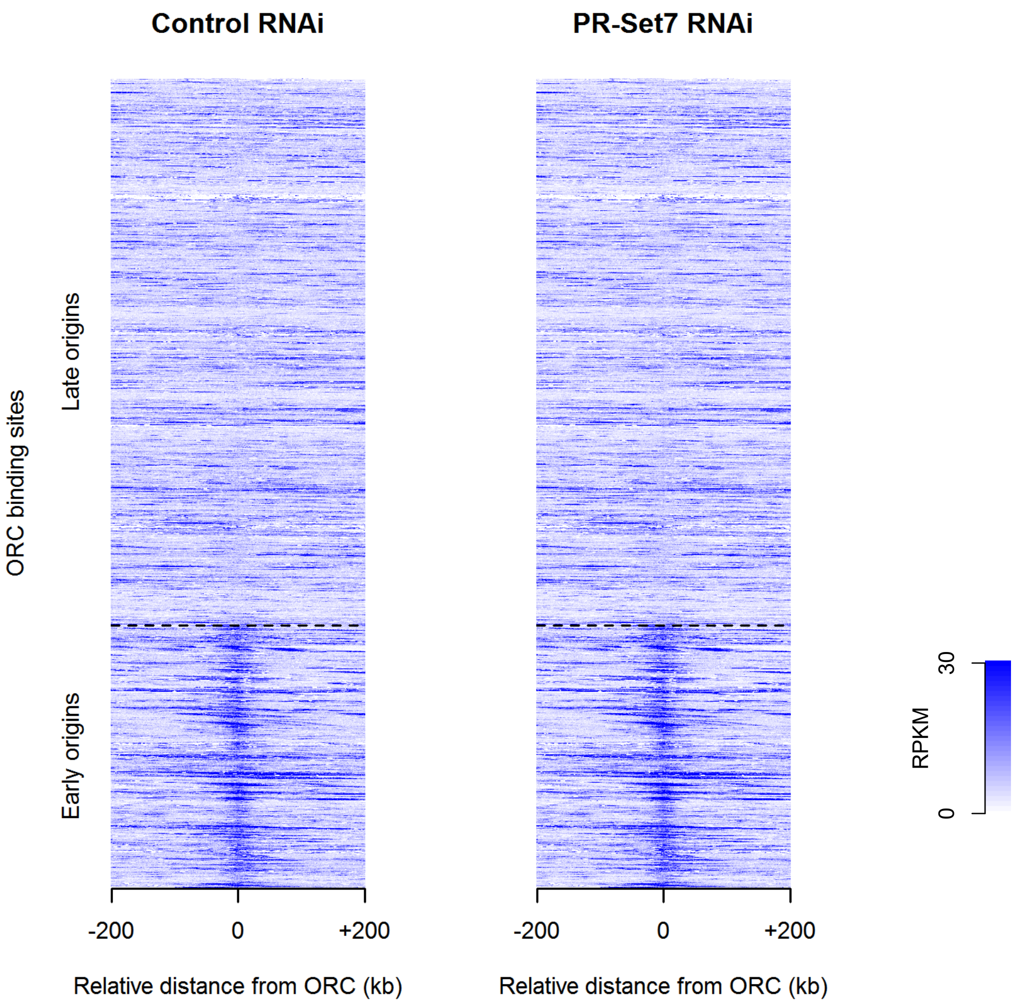
Control RNAi
PR-Set7 RNAi
ORC binding sites
Late origins
Early origins
-200
0
+200
Relative distance from ORC (kb)
-200
0
+200
Relative distance from ORC (kb)
30
RPKM
0

Li et al., Fig S6

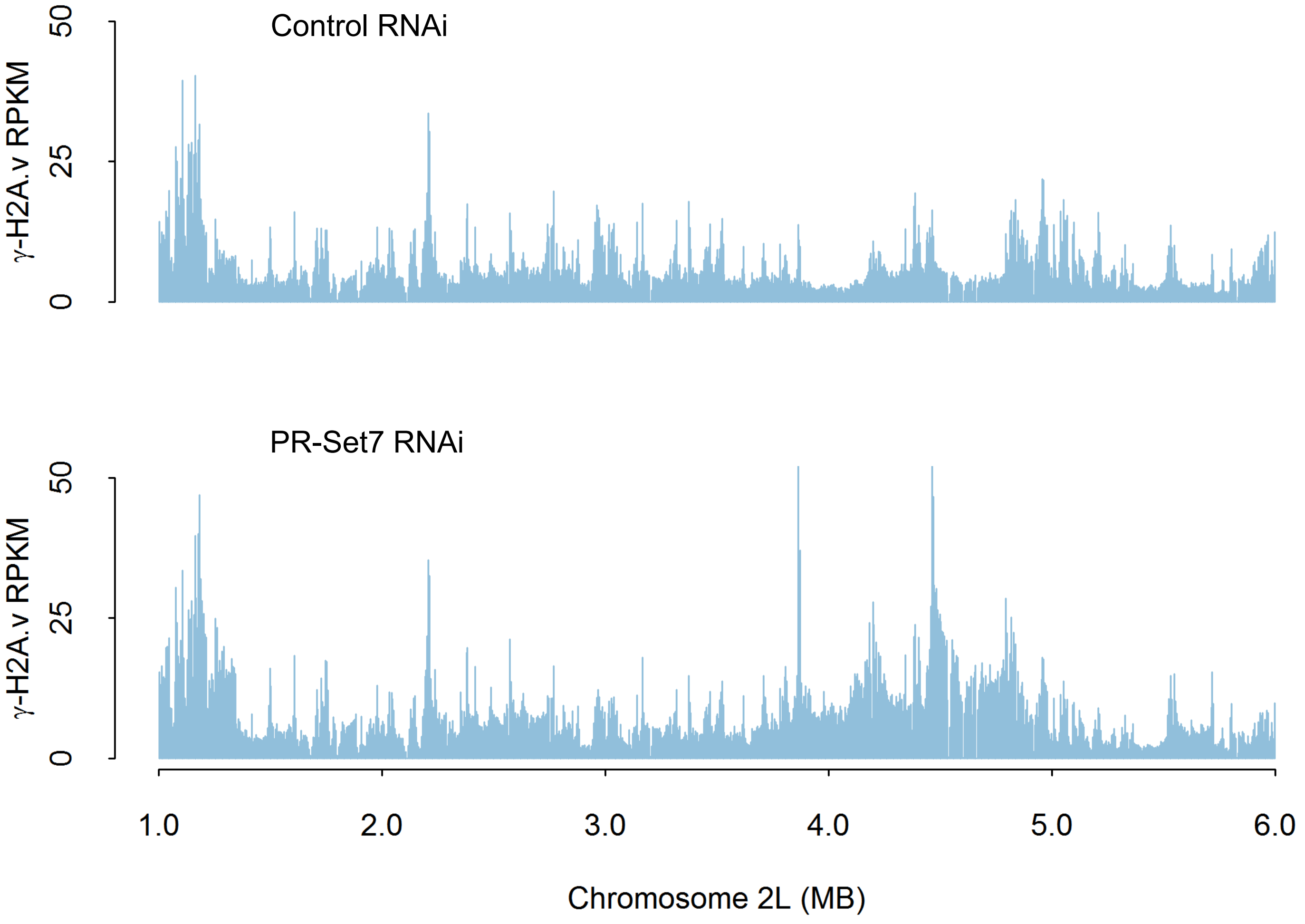

Li et al., Fig S7

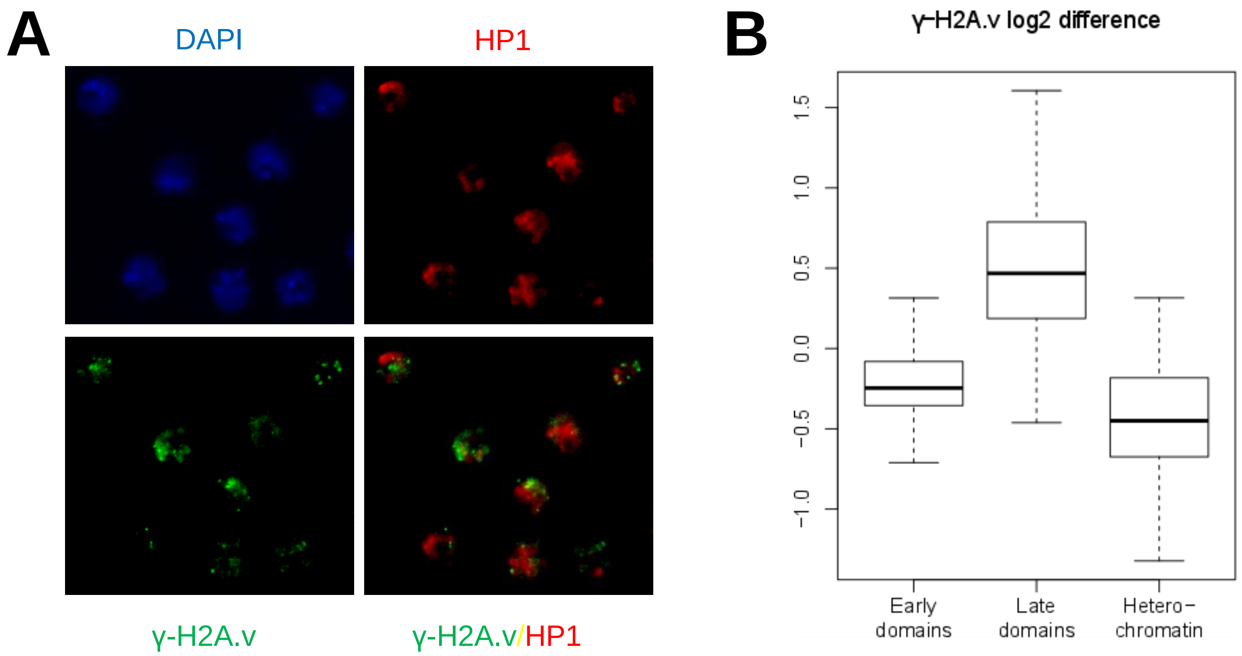

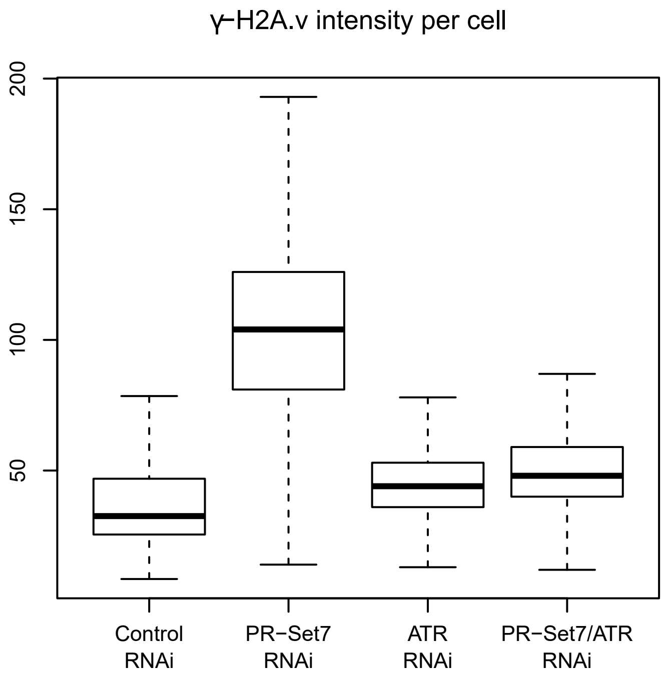
γ−H2A.v intensity per cell
200
150
100
50
Control RNAi
PR−Set7 RNAi
ATR RNAi
PR−Set7/ATR RNAi

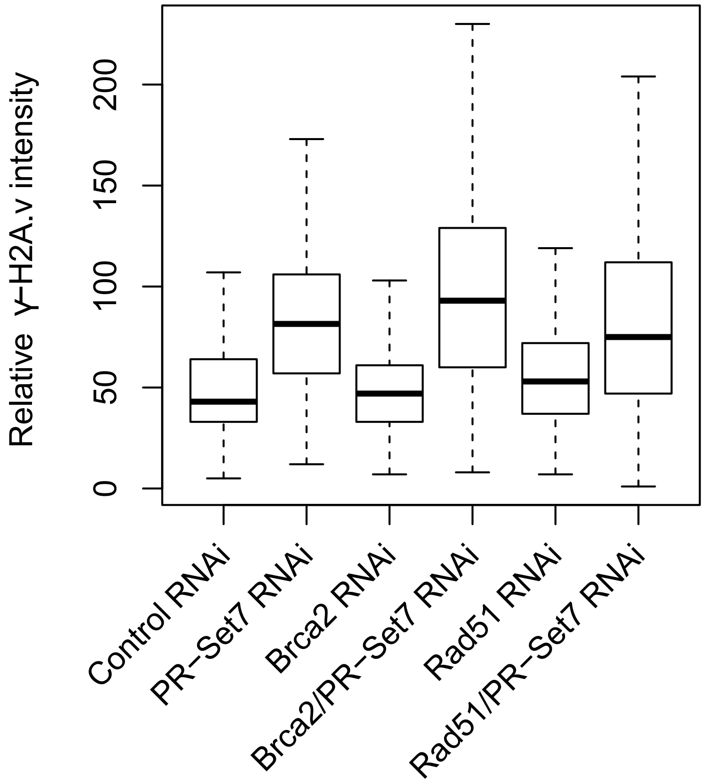
Relative γ-H2A.v intensity
0
50
100
150
200
Control RNAi
PR-Set7 RNAi
Brca2 RNAi
Brca2/PR-Set7 RNAi
Rad51 RNAi
Rad51/PR-Set7 RNAi

Table S1. Chromosomal positions of late replicating domains and γ-H2A.v domains identified by HMM.

	Late replicating domains			γ-H2A.v domains		
	Chr	Start	End	Chr	Start	End
1	2L	627002	813000	2L	580000	810000
2	2L	1005002	1048000	2L	900000	1080000
3	2L	1258002	1350000	2L	1170000	1590000
4	2L	2406002	2648000	2L	1730000	1910000
5	2L	3839002	4200000	2L	2220000	2730000
6	2L	4268002	4326000	2L	3620000	4880000
7	2L	4457002	4793000	2L	5520000	5850000
8	2L	6148002	6294000	2L	6100000	6340000
9	2L	8806002	8864000	2L	7500000	7800000
10	2L	10069002	10138000	2L	10510000	10950000
11	2L	10601002	10842000	2L	11240000	12000000
12	2L	11318002	11787000	2L	12100000	12440000
13	2L	11831002	12002000	2L	13900000	16270000
14	2L	12135002	12362000	2L	16850000	18540000
15	2L	14087002	14178000	2L	19190000	19360000
16	2L	14419002	14486000	2L	19570000	19750000
17	2L	14540002	15011000	2L	19820000	20070000
18	2L	15090002	15239000	2L	20440000	20650000
19	2L	15281002	15654000	2L	21750000	22070000
20	2L	15779002	16221000	2R	2940000	3130000
21	2L	16962002	17339000	2R	4610000	4780000
22	2L	17517002	18105000	2R	9450000	9710000
23	2L	18181002	18409000	2R	11270000	11410000
24	2L	19835002	20110000	2R	12230000	12430000
25	2L	20223002	20372000	2R	13030000	13280000
26	2L	20488002	20573000	2R	14730000	15040000
27	2L	21795002	22112000	2R	15360000	16150000
28	2R	1683002	1791000	2R	16210000	16790000
29	2R	2396002	2454000	2R	17630000	17970000
30	2R	2957002	3086000	2R	18570000	18700000
31	2R	4185002	4322000	2R	18820000	19260000
32	2R	4622002	4799000	3L	1870000	2390000
33	2R	4837002	4972000	3L	2840000	3050000
34	2R	5533002	5724000	3L	3600000	3820000
35	2R	6187002	6296000	3L	4410000	5750000
36	2R	6844002	6899000	3L	5880000	6120000
37	2R	7357002	7423000	3L	6210000	6920000
38	2R	7605002	7723000	3L	7010000	7130000
39	2R	7953002	8008000	3L	7390000	7730000
40	2R	9524002	9679000	3L	9130000	9340000
41	2R	10534002	10641000	3L	9850000	10640000
42	2R	10910002	10965000	3L	11790000	12040000

43	2R	11280002	11369000	3L	13030000	13260000
44	2R	11457002	11670000	3L	13360000	13920000
45	2R	12245002	12425000	3L	14180000	14480000
46	2R	13828002	13889000	3L	14780000	14980000
47	2R	14057002	14157000	3L	15300000	15490000
48	2R	14750002	15022000	3L	15690000	15950000
49	2R	15414002	15580000	3L	16230000	16350000
50	2R	15667002	16094000	3L	17020000	17430000
51	2R	16244002	16491000	3L	18100000	18670000
52	2R	16552002	16869000	3L	19080000	19390000
53	2R	17696002	17930000	3L	20530000	20710000
54	2R	18120002	18181000	3L	21670000	21820000
55	2R	18710002	18761000	3L	21940000	22260000
56	2R	18851002	18929000	3L	22400000	22720000
57	2R	18972002	19237000	3R	1610000	2200000
58	2R	19322002	19435000	3R	2270000	2580000
59	2R	20278002	20386000	3R	3030000	3740000
60	3L	1981002	2147000	3R	4170000	4360000
61	3L	2203002	2350000	3R	6700000	7030000
62	3L	2686002	2731000	3R	7830000	8070000
63	3L	2862002	3064000	3R	8540000	8820000
64	3L	3640002	3760000	3R	8860000	9150000
65	3L	3955002	4008000	3R	9210000	9400000
66	3L	4720002	5094000	3R	10710000	10920000
67	3L	5381002	5569000	3R	12940000	13440000
68	3L	5838002	5887000	3R	13550000	13880000
69	3L	5947002	6068000	3R	14500000	15590000
70	3L	6293002	6878000	3R	15700000	16110000
71	3L	7017002	7113000	3R	16140000	16370000
72	3L	7156002	7234000	3R	17890000	18200000
73	3L	7446002	7716000	3R	18610000	18870000
74	3L	9204002	9293000	3R	19170000	19550000
75	3L	9921002	10203000	3R	21120000	21340000
76	3L	10508002	10582000	3R	21970000	22630000
77	3L	11835002	12045000	3R	23100000	24630000
78	3L	12586002	12663000	3R	24750000	24860000
79	3L	13060002	13187000	3R	25100000	25480000
80	3L	13573002	13799000	3R	26390000	26570000
81	3L	14310002	14370000	3R	26850000	27030000
82	3L	14846002	14962000	3R	27270000	27410000
83	3L	15358002	15418000	3R	27630000	27770000
84	3L	15740002	15786000	X	2660000	2910000
85	3L	15851002	15908000	X	3070000	3180000
86	3L	16247002	16330000	X	4590000	5310000
87	3L	17087002	17437000	X	6250000	6430000
88	3L	18201002	18584000	X	7210000	7410000
89	3L	19166002	19226000	X	9240000	9390000

90	3L	19294002	19580000	X	9580000	10130000
91	3L	20584002	20758000	X	10770000	11130000
92	3L	20862002	20970000	X	11910000	12400000
93	3L	21056002	21125000	X	12670000	13020000
94	3L	21644002	21805000	X	13310000	13470000
95	3L	21977002	22246000	X	13880000	14920000
96	3L	22453002	22702000	X	15020000	15190000
97	3L	24280002	24398000	X	17860000	18140000
98	3R	1667002	2164000	X	19800000	20280000
99	3R	2332002	2586000	X	20370000	20910000
100	3R	2650002	2839000	X	21320000	21430000
101	3R	3059002	3300000	X	21620000	21700000
102	3R	3400002	3594000	X	21910000	22440000
103	3R	3635002	3710000			
104	3R	6260002	6576000			
105	3R	6732002	6982000			
106	3R	7107002	7346000			
107	3R	7923002	8153000			
108	3R	8361002	8424000			
109	3R	8600002	8800000			
110	3R	8918002	9068000			
111	3R	9207002	9408000			
112	3R	9667002	9767000			
113	3R	10755002	10937000			
114	3R	11566002	11609000			
115	3R	12317002	12449000			
116	3R	12491002	12785000			
117	3R	12983002	13191000			
118	3R	13271002	13337000			
119	3R	13648002	13966000			
120	3R	15055002	15413000			
121	3R	15867002	16066000			
122	3R	16176002	16367000			
123	3R	17910002	18226000			
124	3R	18634002	18819000			
125	3R	19224002	19471000			
126	3R	20204002	20304000			
127	3R	20776002	20826000			
128	3R	20971002	21039000			
129	3R	21187002	21263000			
130	3R	22132002	22244000			
131	3R	22390002	22522000			
132	3R	23177002	23375000			
133	3R	23428002	23735000			
134	3R	23900002	24048000			
135	3R	24195002	24338000			
136	3R	24481002	24610000			

137	3R	24720002	24828000			
138	3R	25210002	25529000			
139	3R	26316002	26505000			
140	3R	26910002	27013000			
141	X	2747002	2791000			
142	X	3910002	3981000			
143	X	4301002	4409000			
144	X	4652002	4817000			
145	X	4892002	5170000			
146	X	5900002	6067000			
147	X	7284002	7375000			
148	X	8190002	8255000			
149	X	9273002	9387000			
150	X	9630002	10122000			
151	X	10768002	10955000			
152	X	11994002	12289000			
153	X	13375002	13448000			
154	X	13954002	14078000			
155	X	14204002	14532000			
156	X	14588002	14654000			
157	X	15058002	15157000			
158	X	17172002	17370000			
159	X	17811002	17949000			
160	X	18028002	18144000			
161	X	18859002	18915000			
162	X	19852002	20037000			
163	X	20101002	20249000			
164	X	20423002	20877000			
165	X	21335002	21456000			
166	X	22028002	22211000			

Table S2. Primer sequences used for generating dsRNAs.

dsRNA Target	Forward Primer	Reverse Primer
PR-Set7	CACAATGATGGCACACAACA	TTCGAAGCTCCGATTGATCT
Suv4-20	CTACGGCGAGGACTTCTTTG	CTTTTCGAGCAAAGAGACGG
ATR	CGACATGGACACAAAACTGG	TTTGGGTAAGCACTTGGACC
ATM	GCGTTCTGCTGGAAGATG	GCTCATCCAAACTAGCGTAA
Rad51	CTCGCACCTTCTATCAAATG	CATCGGCCAGGCGTTG
Brca2	CGACGAAATGGCCAAAATAT	CCTGCCTTGCTTCGGTT
Control (pUC119)	GCGCGGAACCCCTATTTGTTTA	GGAAGCTAGAGTAAGTAGTTCGC

Experiment	Sample	Replicate #	Pearson correlation
H4K20me1 ChIP-seq	HU arrest	1	0.949
	sorted G1/S	1	
	HU release	1	0.901
	sorted late S/G2	1	
Early origin	control RNAi	1	#1, #2: 0.898
		2	#2, #3: 0.921
		3	#1, #3: 0.958
	PR-Set7 RNAi	1	0.847
		2	
	ATR RNAi	1	0.726
		2	
	ATR/PR-Set7 RNAi	1	0.876
		2	
	mock treatment	1	
	Suv4-20 RNAi	1	0.966*
	PR-Set7/Suv4-20 RNAi	1	
	PR-Set7 o/e	1	0.953*
	PR-Set7 o/e + Suv4-20 RNAi	1	
γ-H2A.v ChIP-seq	control RNAi	1	0.974
		2	
	PR-Set7 RNAi	1	0.975
		2	

* Samples are genetically redundant, so they could serve as biological replicates.
